# Therapeutic Insights into *Rubus ulmifolius* Schott Leaf Extract: In Vitro Antifungal, Enzyme Inhibition, and Anticancer Activities Integrated with Network Pharmacology and Molecular Docking Analyses of Colorectal and Ovarian Cancer

**DOI:** 10.3390/ph18101563

**Published:** 2025-10-16

**Authors:** Amina Bramki, Ghozlane Barboucha, Ouided Benslama, Fatiha Seglab, Fatima Zohra Makhlouf, Sirine Nessah, Chawki Bensouici, Marco Masi, Abdullah A. Shaito

**Affiliations:** 1 Higher National School of Biotechnology, Taoufik Khaznadar, Nouveau Pôle Universitaire Ali Mendjeli, BP E66, Constantine 25100, Algeria; a.bramki@ensbiotech.edu.dz (A.B.); g.barb@ensbiotech.edu.dz (G.B.); makhlouf_f.zohra@umc.edu.dz (F.Z.M.); sirinsirin2000hh@gmail.com (S.N.); 2 Department of Natural and Life Sciences, Faculty of Exact Sciences and Natural and Life Sciences, Larbi Ben M’Hidi University, Oum El Bouaghi 04000, Algeria; ouided.benslama@univ-oeb.dz; 3 Biomedical Research Center (BRC), QU Health, Qatar University, Doha P.O. Box 2713, Qatar; f.seglab@gmail.com; 4 Biotechnology Research Center, UV 03, BP. E73, Ali Mendjeli, Constantine 25016, Algeria; c.bensouici@crbt.dz; 5 Department of Chemical Sciences, University of Naples Federico II, 80126 Naples, Italy; 6 Biomedical Sciences Department, College of Health Sciences, QU Health, Qatar University, Doha P.O. Box 2713, Qatar; 7 College of Medicine, QU Health, Qatar University, Doha P.O. Box 2713, Qatar

**Keywords:** *Rubus ulmifolius* Schott, GC-MS, antifungal activity, AChE, BChE, urease enzyme inhibition, anticancer activity, network pharmacology, molecular docking

## Abstract

**Background/Objectives:** This study evaluated the antifungal, enzyme inhibitory, and anticancer properties of the ethyl acetate (EtOAc) leaves extract of *Rubus ulmifolius* Schott using in vitro assays and in silico analysis. **Methods:** Antifungal activity was assessed against five fungal strains by measuring inhibition zones. Enzyme inhibition assays were conducted for acetylcholinesterase (AChE), butyrylcholinesterase (BChE), and urease. Antiproliferative effects were tested against HT-29 colorectal, SK-OV-3 ovarian, and A549 lung cancer cells using the MTT assay. Network pharmacology and molecular docking analyses were performed on major compounds previously identified by GC–MS (gallic acid, caffeic acid, catechin, and fructofuranose) to uncover the potential mechanisms of the plant in colorectal and ovarian cancers. **Results:** The extract displayed notable antifungal activity, particularly against *Penicillium* sp., *Aspergillus fumigatus*, and *Candida albicans*, with inhibition zones of 22.5 ± 0.7 to 26.8 ± 1.3 mm. Enzyme assays revealed moderate inhibition of AChE (IC_50_ = 92.94 ± 1.97 µg/mL), weaker activity against BChE (IC_50_ = 274.93 ± 2.32 µg/mL), and modest inhibition of urease (IC_50_ = 262.60 ± 1.41 µg/mL). The extract exhibited strong antiproliferative effects against HT-29 and SK-OV-3 cells (IC_50_ = 2.41 ± 0.13 and 4.63 ± 0.26 µg/mL, respectively), whereas activity against A549 lung cancer cells was limited. Network pharmacology predicted 52 and 44 overlapping target genes between the major compounds and colorectal and ovarian cancers, respectively. Protein–protein interaction networks identified hub genes for each cancer type, with key shared targets including *EGFR*, *ESR1*, *PTGS2*, and *STAT3*. Molecular docking confirmed favorable binding between these targets and the compounds, particularly catechin, which showed interactions comparable to those of reference inhibitors. **Conclusions:** These findings suggest that *R. ulmifolius* may possess multi-target antifungal, neuroprotective, and anticancer potential, warranting further in vitro pharmacological and preclinical validation.

## 1. Introduction

In recent years, modern medicine has increasingly focused on harnessing therapeutics derived from natural resources, particularly traditionally used medicinal plants. Their medicinal value lies in the abundance of phytochemicals, secondary metabolites, with diverse biological activities such as antioxidant, antimicrobial, antitumor, and anti-inflammatory properties, among others [1,2,3,4]. With the rising global demand for bioactive natural products in the pharmaceutical, cosmetic, and other industries, the discovery of novel therapeutics from natural sources, including plant-based therapeutics, has become a significant research priority alongside conventional drug development [3,5,6]. This need is further emphasized by current global health challenges, including epidemic diseases [7].

Among medicinal plants, the *Rubus* genus (Rosaceae family) stands out as one of the most extensive and diverse plant groups, comprising over 750 species distributed worldwide [8]. While often cultivated for their edible fruits [9], several *Rubus* species have long been used in traditional medicine to alleviate a variety of ailments [10]. The significant medicinal value of *Rubus* species is largely attributed to their high content of flavonoids, tannins, anthocyanins, and phenolic acids [11]. *Rubus ulmifolius* Schott is a perennial species native to Europe and North America [9], and now widely distributed across diverse regions, including North Africa, parts of Asia [12], and South American countries such as Brazil [9]. It grows naturally in hedgerows, forest edges, and fallow lands, contributing to the maintenance of the rural ecosystem [1].

In traditional folk medicine, various parts of *R. ulmifolius* have been used to treat a range of ailments. The leaves and roots are used to treat skin disorders, while in Italian traditional medicine, the plant has been applied to manage abscesses, ulcers, and diarrhea, and is used externally as a vaginal wash [11]. Its fruits, consumed for their health benefits, are reported to have antioxidant and anti-inflammatory properties [9]. The plant reportedly has versatile biological properties, such as antioxidant [9,13,14,15,16], antimicrobial [1,12,15,17,18,19], antidiabetic [11], and cytotoxic/anticancer effects, among other activities [12,15,20].

Despite these reported effects, the therapeutic potential of this species remains largely underexplored. To address this gap, the present study evaluated, for the first time, the antifungal activity of the leaf extract, the enzyme inhibitory effects against acetylcholinesterase (AChE), butyrylcholinesterase (BChE), and urease, as well as the anticancer potential of the ethyl acetate (EtOAc) extract through both in vitro assays and in silico analyses.

The antiproliferative activity was assessed against human lung adenocarcinoma (A549), colorectal adenocarcinoma (HT-29), and ovarian cancer (SK-OV-3) cell lines using the MTT assay.

Furthermore, integrative network pharmacology and molecular docking analyses were conducted to elucidate the mechanisms underlying its anticancer activity, particularly against colorectal and ovarian cancer. These computational strategies provide a systems-level perspective by linking bioactive compounds to disease-associated gene targets, enabling the identification of key pathways and therapeutic targets [21]. The in silico workflow incorporated compound–target prediction using SwissTargetPrediction (Version 2025) and Similarity Ensemble Approach (SEA) tools (Version 2025), disease–gene association mapping (GeneCards, OMIM), protein–protein interaction (PPI) network construction, KEGG functional pathway enrichment, and molecular docking. This integrative approach offers novel insights into the pharmacological relevance of *R. ulmifolius* metabolites and highlights its potential as a promising source of plant-based therapeutic agents.

## 2. Results and Discussion

### 2.1. R. ulmifolius Leaves EtOAc Extract Exhibits Antifungal Activities

The results of the antifungal activity assay showed that the EtOAc extract exhibited a significant antifungal effect against all of the tested fungal isolates compared to nystatin (Table 1). The highest inhibition zones were observed against *Penicillium* sp., *A. fumigatus*, and *C. albicans*, with diameters ranging from 26.8 ± 1.3 to 22.5 ± 0.7 mm, followed by moderate effects against *F. oxysporum* and *A. niger*, with diameters of 18.0 ± 1.0 mm each (Table 1).

These results are consistent with those reported by Sisti et al. [22], who evaluated the antifungal activity of the methanolic extract and phenolic fractions of *R. ulmifolius* seedlings aerial parts against 37 fungal strains. Their study revealed inhibition zones with diameters ranging from 7.7 ± 0.7 to 29.0 ± 1 mm. These values are higher than those reported by Panizzi et al. [17], who investigated organic extracts from the aerial parts (leaves, stems, and flowering tops) of *R. ulmifolius* against *Saccharomyces cerevisiae*, *C. albicans*, and *A. niger*, and found inhibition zone diameters ranging from 8.1 to 20 mm. GC-MS analysis revealed several low molecular weight constituents in the EtOAc extract [23], including gallic acid, caffeic acid, catechin, and D-(-)-Fructofuranose. In the study by Bramki et al. [23], *R. ulmifolius* EtOAc extract was derivatized, following the method described by Guida et al. [24], to identify the silylated surrogates of the polar low molecular weight compounds, in addition to the polyaromatic hydrocarbons, and volatile and thermally stable compounds that can be analyzed directly by GC-MS. Of note, an HPLC-based separation technique should be used when the aim is to identify more polar and higher molecular weight compounds, including phenolic compounds and flavonoids [25,26].

Gallic acid, caffeic acid, and catechin are likely contributors to *R. ulmifolius* antifungal activity. Conversely, D-(-)-Fructofuranose, being a monosaccharide, is not known to exhibit antifungal properties. According to El-Nagar et al. [27], gallic acid and its derivatives exhibit notable in vitro fungistatic effects against *Alternaria solani*. Similarly, Akhtar et al. [28] have reported gallic acid as a key antifungal component in the leaf extract of *Sarcochlamys pulcherrima*, with confirmed activity against *Candida auris* and *C. albicans*. Both in vitro and in silico analyses suggested its ability to inhibit fungal growth by targeting carbonic anhydrase enzymes. Caffeic acid and its derivatives have also been reported to display fungicidal activity against *C. albicans* [29]. In addition, Hirasawa and Takada [30] showed that catechin exhibits antifungal effects against *C. albicans*, providing preliminary evidence that these phenolic compounds contribute to the extract’s antifungal properties. These findings suggest that *R. ulmifolius* leaf extracts, through their phenolic constituents, could serve as a promising starting point for the development of plant-based antifungal agents.

### 2.2. R. ulmifolius Leaves EtOAc Extract Has a Moderate AChE and BChE Inhibitory Activity

The inhibitory effects of the EtOAc extract on AChE and BChE are presented in Table 2. Compared to the reference inhibitor galantamine, the extract showed moderate inhibition of AChE and weak inhibition of BChE.

AChE and BChE are key enzymes responsible for the hydrolysis of the neurotransmitter acetylcholine. Their overactivity is closely associated with the progression of Alzheimer’s disease [31,32]. Consequently, targeting these enzymes, especially through natural products, has become a promising area of interest due to the favorable safety profiles and accessibility of plant-derived compounds. While no previous studies have specifically investigated the cholinesterase inhibitory potential of *R. ulmifolius*, related species within the *Rubus* genus have been reported to display comparable bioactivity. For instance, Saqee and Eruygur [33] investigated the enzyme inhibitory activity of methanolic fruit extracts of *R. fruticosus* and reported moderate AChE and BChE inhibition when compared to galantamine. In contrast, Yener [34] found that ethanolic extracts of *R. sanctus* exhibited notably strong inhibitory effects for both enzymes, even approaching or surpassing galantamine in some assays. Such differences across studies could be related to variations in species, extraction solvents, and phytochemical composition of the extracts [35,36]. The moderate cholinesterase inhibition observed in the present study may be attributed to the extract’s phytochemical composition, particularly the presence of gallic acid, caffeic acid, and catechin, which have been reported to display mild cholinesterase inhibitory activity [37,38]. In contrast, D-(-)-Fructofuranose, a simple sugar, is not known to exhibit inhibitory activity against cholinesterase enzymes. Several studies have reported that polyphenolic compounds exhibit moderate to low inhibitory capacity against cholinesterases. Catechin, for instance, has demonstrated mild activity in AChE inhibition assays, with a generally low but occasionally detectable affinity toward AChE [38,39]. The cholinesterase-inhibiting potential of gallic acid and caffeic acid is similarly modest, with effective inhibition generally requiring relatively high concentrations of the compounds [40,41]. Although none of these individual compounds is considered a potent inhibitor, their combined presence in the EtOAc extract could exert additive or synergistic effects [42], contributing to the overall observed inhibitory potential. Interestingly, the selectivity observed in the present study, with a higher inhibition of AChE compared to BChE, might be of particular interest in the context of Alzheimer’s disease progression. AChE plays a more dominant role in the early stages of the disease, whereas BChE becomes increasingly important in later stages [43,44]. Therefore, selective AChE inhibition, such as that observed with the *R. ulmifolius* extract, provides preliminary evidence that may support further studies on its potential benefits in preventive strategies or early-stage therapeutic interventions.

### 2.3. R. ulmifolius Leaves EtOAc Extract Has a Modest Urease Inhibitory Assay

Urease is a nickel-dependent metalloenzyme that catalyzes the hydrolysis of urea into ammonia and carbon dioxide. Its ureolytic activity is a major contributor to the pathologies induced by *Helicobacter pylori* and *Proteus* species [45,46]. Consequently, urease inhibition has gained therapeutic importance due to its potential in treating various conditions such as gastric ulcers, urinary tract infections, and hepatic encephalopathy [47,48]. In the present study, *R. ulmifolius* leaves EtOAc extract exhibited less potent inhibitory activity than the standard inhibitor thiourea (IC_50_ 11.57 ± 0.68 µg/mL). However, the extract’s urease inhibitory activity (IC_50_ 262.60 ± 1.41 µg/mL) is considered moderate (Table 3) and may have a therapeutic value.

The modest activity observed in *R. ulmifolius* may be attributed to its phenolic constituents, including gallic acid, caffeic acid, and catechin. These compounds have been reported to inhibit urease through hydrogen bonding or chelation of the nickel ions essential for enzymatic activity [47,49]. For instance, catechin and gallic acid have demonstrated mild to moderate urease inhibition, typically at relatively high concentrations [50]. Similarly, caffeic acid has been identified as a non-competitive urease inhibitor in the ethyl acetate fraction of *Taraxacum officinale* with an IC_50_ around 184 µg/mL [51]. Thus, the moderate effect observed in the present study could also result from synergistic interactions among multiple phenolic constituents, as commonly reported in plant extracts [52]. To our knowledge, this is the first report of a urease inhibitory activity in *R. ulmifolius*. More broadly, this is one of the very few studies addressing urease inhibitory activity within the *Rubus* genus. A notable exception is the study by Yang et al. [53], which identified a proteinaceous urease inhibitor from *R. coreanus* with strong inhibitory activity against *H. pylori* urease (IC_50_ = 19.4 ± 0.43 μM). Although structurally distinct from the phenolics identified in our extract, this finding suggests that *Rubus* species are capable of biosynthesizing compounds with potential urease inhibitory activity.

### 2.4. R. ulmifolius Leaves EtOAc Extract Has a Potent Cytotoxic Activity Against Cancerous Human Cell Lines

**Figure 1:**
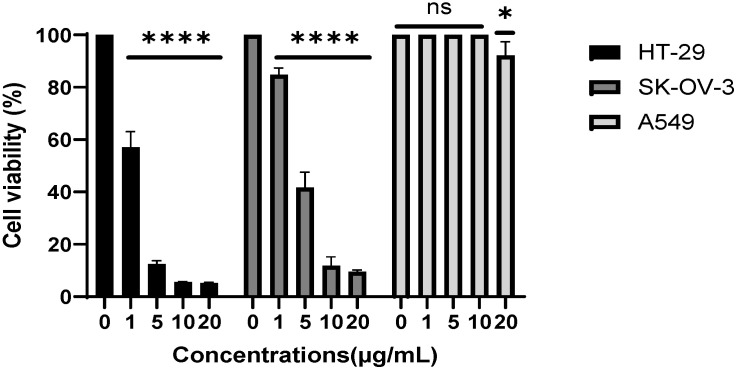
Effect of *R. ulmifolius* EtOAc extract on the viability of three human cancer cell lines: A549, HT-29, and SK-OV-3. Cells were treated with the indicated concentrations of the extract for 48 h and subjected to the MTT assay. The results represent the percentage cell viability of the treated cells compared to the viability of vehicle-treated cells, which was set as 100%. Results are expressed as mean ± SD of 3 independent experiments (n = 3). Statistical significance was determined compared to the control group (* *p* < 0.05, **** *p* < 0.0001; ns = not significant).

The antiproliferative effects of the EtOAc extract against human cancerous cell lines HT-29 (colorectal adenocarcinoma), SK-OV-3 (ovarian cancer), and A549 (lung adenocarcinoma) are presented in both Table 4 and Figure 1.

The EtOAc extract exhibited a notable cytotoxic effect against the HT-29 and SK-OV-3 cancer cell lines, with IC_50_ values of 2.41 ± 0.13 and 4.63 ± 0.26 µg/mL, respectively (Table 4 and Figure 1), suggesting a marked antiproliferative potential. According to the NCI guidelines, a crude extract is considered to exhibit significant cytotoxic activity if it shows an IC_50_ value of less than 20 µg/mL [54,55]. Based on this classification, the EtOAc extract can be regarded as cytotoxic against both the HT-29 and SK-OV-3 cell lines. In contrast, the extract was less cytotoxic to A549 lung cancer cells. The cells were only slightly affected by the tested EtOAc concentrations, with a mere 7.79% reduction in cell viability being observed at the highest tested concentration of 20 µg/mL (92.1% cell viability). This suggests that higher extract concentrations or prolonged treatment may be required to achieve a more pronounced cytotoxic effect against A549 cells. To the best of our knowledge, the cytotoxic and anticancer properties of *R. ulmifolius* remain relatively unexplored, with only a limited number of studies addressing these biological activities, and no study has investigated the cytotoxic potential of its EtOAc extract. Among the few available studies, some have nonetheless reported interesting antiproliferative effects. For instance, Triggiani et al. [56] reported that an ethanolic leaf extract of *R. ulmifolius* showed a dose-dependent antiproliferative effect on murine myeloma cells (P3X63-Ag8.653), suggesting the presence of active cytotoxic compounds. Complementary findings were reported by Da Silva et al. [57], who focused on a rich anthocyanin extract derived from *R. ulmifolius* fruits. Their results indicated antiproliferative effects against several human cancer cell lines, including HeLa (cervical carcinoma), HepG2 (hepatocellular carcinoma), MCF-7 (breast adenocarcinoma), and NCI-H460 (non-small cell lung cancer). Among these, HepG2 cells appeared to be the most sensitive (IC_50_ = 286 ± 13 µg/mL), whereas NCI-H460 cells were more resistant (IC_50_ = 337 ± 11 µg/mL) [57]. Despite the slightly high IC_50_ values, the findings provide preliminary evidence of the extract’s potential to suppress tumor cell proliferation. Results of the study by Rodrigues et al. [20] further support *R. ulmifolius* anticancer potential, where the extract displayed moderate cytotoxicity against HT-29, SiHa (cervical cancer), and HeLa cells with IC_50_ values of 294 ± 26, 290 ± 61, and 254 ± 35 μg/mL, respectively [20].

Notably, *R. ulmifolius* extracts have been reported to exhibit minimal toxicity in non-tumorigenic cell models, indicating the selectivity of the plant extracts towards cancer cells. Low cytotoxicity was observed in PLP2 liver cells (IC_50_ > 400 µg/mL) [57], and only minimal effects were detected in normal murine fibroblasts (L929, IC_50_ > 1000 µg/mL) [20]. More recently, low cytotoxicity was also reported in macrophages, with a high selectivity index (SI = 20.67), indicating preferential activity against cancer cells [58].

The EtOAc extract main constituents included D-(-)-fructofuranose, gallic acid, caffeic acid, and catechin, as revealed by GC-MS [23]. The potent antiproliferative activity observed is likely due to the latter three bioactive compounds. Gallic acid has been previously studied for its cytotoxic effects against a wide range of cancer cell lines, such as lung, prostate, breast, leukemia, gastric, colon, cervical, esophageal [59], and liver cancer cells [60]. Recent findings suggest that its anticancer effects are mediated through several biological mechanisms, including the inhibition of cell migration and metastasis [61], induction of apoptosis, cell cycle arrest, and modulation of oncogene expression [62]. In addition, caffeic acid may also contribute to the observed antiproliferative activity. Several studies have highlighted its potential, attributing its effects to various cellular responses initiated upon treatment. Notably, it has been shown to display antiproliferative, pro-apoptotic, and anti-invasive effects in HCT116 colorectal cancer cells by altering the expression of genes and microRNAs involved in apoptosis, cell-cycle regulation, and invasion, while also reducing oxidative stress and glutathione S-transferase (GST) activity [63]. Similarly, caffeic acid treatment has been reported to reduce cell viability, inhibit colony formation, modulate the cell cycle, induce apoptosis, and alter the expression of caspase genes in SK-Mel-28 cells [64]. Moreover, catechin and its derivatives have been shown to exert various anticancer effects; it has been demonstrated to inhibit A549 cell proliferation by inducing cell cycle arrest through the p21 pathway and reducing cyclin E1 and AKT phosphorylation, suggesting a possible antitumor potential [65]. In addition, (+)-catechin isomer has shown significant anti-angiogenic and anti-inflammatory effects in both in vivo (B16F-10 melanoma model in mice) and in vitro (endothelial cells and macrophage assays). Its mechanism was suggested to involve inhibition of endothelial cell proliferation and migration, along with the downregulation of key pro-inflammatory and pro-angiogenic mediators such as TNF-α, nitric oxide, and VEGF, which are essential for tumor-induced angiogenesis [66]. Additionally, caffeic acid has been noted to enhance the effects of certain chemotherapeutic agents, supporting its potential role in combination therapies [67,68]. These findings provide preliminary support for the potential anticancer activity of *R. ulmifolius* phenolics, which warrants further preclinical investigation

### 2.5. In Silico Analyses of the Anticancer Mechanisms of R. ulmifolius 

#### 2.5.1. Identification of Potential Targets of Bioactive Compounds in Colorectal and Ovarian Cancer

**Figure 2:**
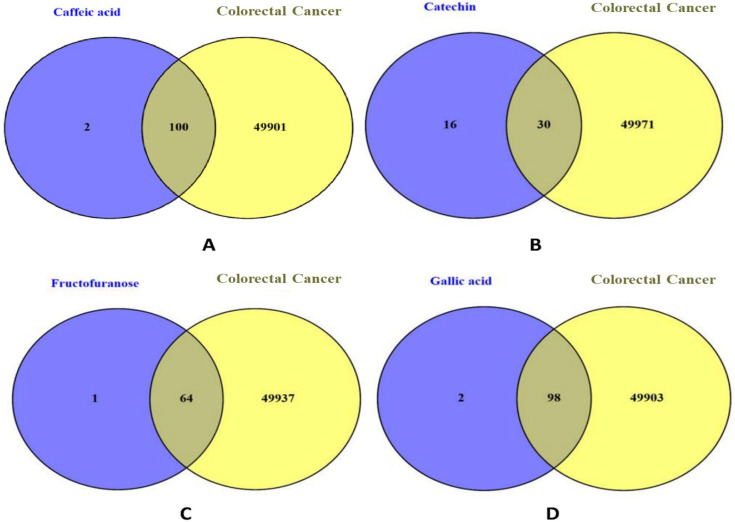
Venn diagrams illustrating the overlap between the predicted molecular targets of individual phytochemical compounds and colorectal cancer-associated genes. (**A**) Overlap between caffeic acid targets and colorectal cancer-related genes. (**B**) Overlap between catechin targets and colorectal cancer-related genes. (**C**) Overlap between fructofuranose targets and colorectal cancer-related genes. (**D**) Overlap between gallic acid targets and colorectal cancer-related genes.

To further investigate the underlying mechanisms of *R. ulmifolius*-mediated antiproliferative activity and the potential therapeutic relevance of the plant extract against two major malignancies, colorectal cancer and ovarian cancer, a two-step in silico strategy was employed: compound-target prediction and disease-gene collection. The molecular targets of the four main bioactive constituents, caffeic acid, catechin, fructofuranose, and gallic acid, were predicted using both the SwissTargetPrediction and Similarity Ensemble Approach (SEA) databases to maximize coverage. For colorectal cancer, the target prediction revealed 101 targets for caffeic acid, 31 for catechin, 65 for fructofuranose, and 99 for gallic acid (Figure 2).

**Figure 3:**
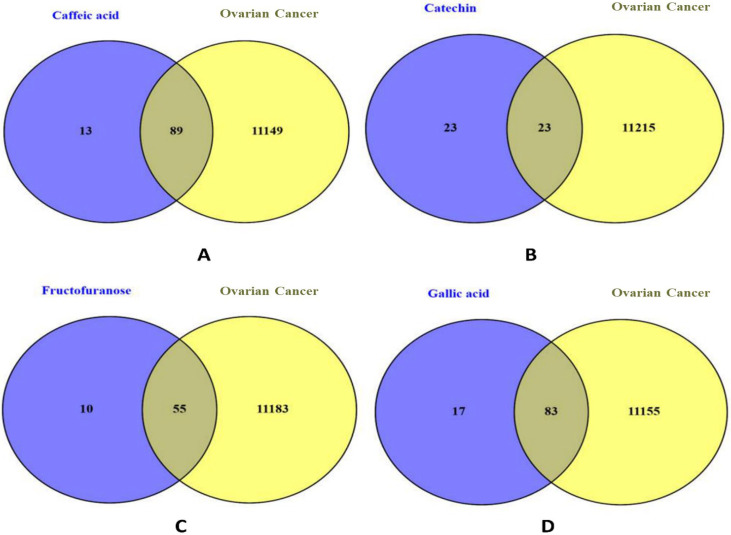
Venn diagrams illustrating the overlap between the predicted molecular targets of individual phytochemical compounds and ovarian cancer-associated genes. (**A**) Overlap between caffeic acid targets and ovarian cancer-related genes. (**B**) Overlap between catechin targets and ovarian cancer-related genes. (**C**) Overlap between fructofuranose targets and ovarian cancer-related genes. (**D**) Overlap between gallic acid targets and ovarian cancer-related genes.

In contrast, for ovarian cancer, the corresponding numbers were slightly different: 90 predicted targets for caffeic acid, 24 for catechin, 56 for fructofuranose, and 84 for gallic acid (Figure 3).

On the disease side, gene sets associated with colorectal cancer (50,001 entries) and ovarian cancer (11,238 entries) were retrieved from GeneCards and OMIM, which include curated data based on gene–disease associations. Venn diagram analyses were then performed for each compound by intersecting its predicted targets with the disease-specific gene sets. This allowed the identification of compound-specific targets potentially relevant to each cancer type. The results are illustrated in Figure 2 for colorectal cancer and Figure 3 for ovarian cancer, highlighting the unique and shared target distributions across the four phytochemicals.

#### 2.5.2. Analysis of PPI and Core Target Networks

**Figure 4:**
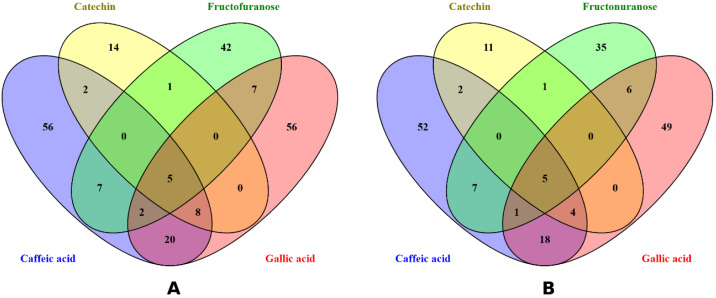
Venn diagram analysis of predicted targets of the plant-derived compounds intersected with disease-related genes. (**A**) Overlapping target genes between the compounds and the colorectal cancer gene set, identifying 52 common targets shared by at least two compounds. (**B**) Overlapping targets with the ovarian cancer gene set, yielding 44 shared genes.

**Figure 5:**
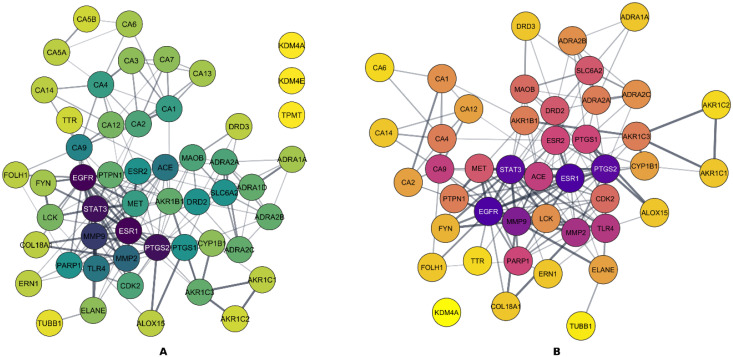
Protein–protein interaction (PPI) networks of the shared targets between bioactive compounds and cancer-related genes. (**A**) PPI network of 52 common targets shared between at least two phytochemicals and colorectal cancer genes. (**B**) PPI network of 44 common targets shared between at least two phytochemicals and ovarian cancer genes. Node color gradients indicating the node degree (connectivity), ranging from dark purple (high-degree) to light yellow (low-degree hub proteins).

To explore the mechanistic relevance of the phytochemical constituents identified in the plant extract, we conducted a protein–protein interaction (PPI) network analysis of the predicted targets intersecting with disease-related genes for both colorectal and ovarian cancers. In the case of colorectal cancer, overlapping phytochemicals-lung cancer targets using Venn diagrams identified 52 common target genes that were shared by at least two of the four compounds (Figure 4A). Similarly, overlapping of Venn diagrams identified 44 such target genes for ovarian cancer (Figure 4B). These overlapping targets (Figure 4) represent key molecular interfaces potentially modulated by the extract and were used to construct the PPI network (Figure 5).

The corresponding PPI networks were constructed using the STRING database and visualized in Cytoscape (Figure 5A,B), revealing interconnected clusters suggestive of functional convergence among cancer-associated pathways.

**Figure 6:**
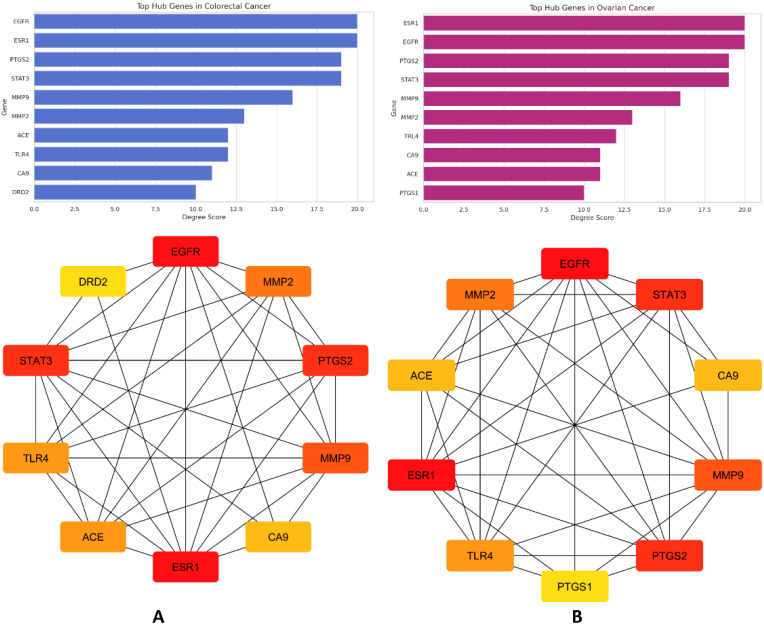
Comparative visualization of hub gene networks and their respective degree scores in colorectal and ovarian cancer. (**A**) Top 10 hub genes identified in colorectal cancer. (**B**) Top 10 hub genes identified in ovarian cancer. Node color represents the degree of connectivity, ranging from darker red color indicating higher degree (more connected hub genes) and lighter yellow indicating lower degree values.

Topological analysis of the PPI networks enabled the identification of core targets, or hub genes, based on degree centrality. For both cancers (Figure 6A,B), a striking convergence was observed in the hub gene profile. Specifically, *EGFR* and *ESR1* were the most prominent nodes, each exhibiting the highest degree score of 20 in both networks. These were followed closely by *PTGS2* and *STAT3*, both of which scored 19 nodes in each condition, highlighting their pivotal roles in inflammatory and proliferative signaling. Matrix metalloproteinases *MMP9* and *MMP2* also appeared as key hubs in both cancers, consistent with their well-established involvement in extracellular matrix remodeling and cancer metastasis. Additional shared targets included *ACE*, *TLR4*, and *CA9*, while *DRD2* was uniquely present among the top colorectal cancer targets, and *PTGS1* appeared exclusively in ovarian cancer.

This high degree of overlap in hub targets suggests that the phytochemical constituents may exert broad-spectrum anticancer effects through conserved molecular pathways. The presence of inflammation-related mediators (e.g., *PTGS2*, *TLR4*), hormone receptors (e.g., *ESR1*), and key growth and survival regulators (e.g., *EGFR*, *STAT3*) among the top hubs highlights the multi-target potential of the extract, possibly enabling it to modulate critical oncogenic networks shared across cancer types. These findings provide a strong rationale for the subsequent exploration of binding affinities through molecular docking analyses.

#### 2.5.3. KEGG Enrichment of Core Targets

The KEGG pathway enrichment analysis [69] of the top 10 hub genes revealed key biological processes and signaling cascades associated with both colorectal and ovarian cancers. Notably, both cancer types shared several enriched pathways, including pathways in cancer, proteoglycans in cancer, endocrine resistance, estrogen signaling, and microRNAs in cancer. These commonalities suggest that, despite the distinct tissue origins, core regulatory mechanisms involving hormone signaling, transcriptional control, and epigenetic regulation play crucial roles in both malignancies.

**Figure 7:**
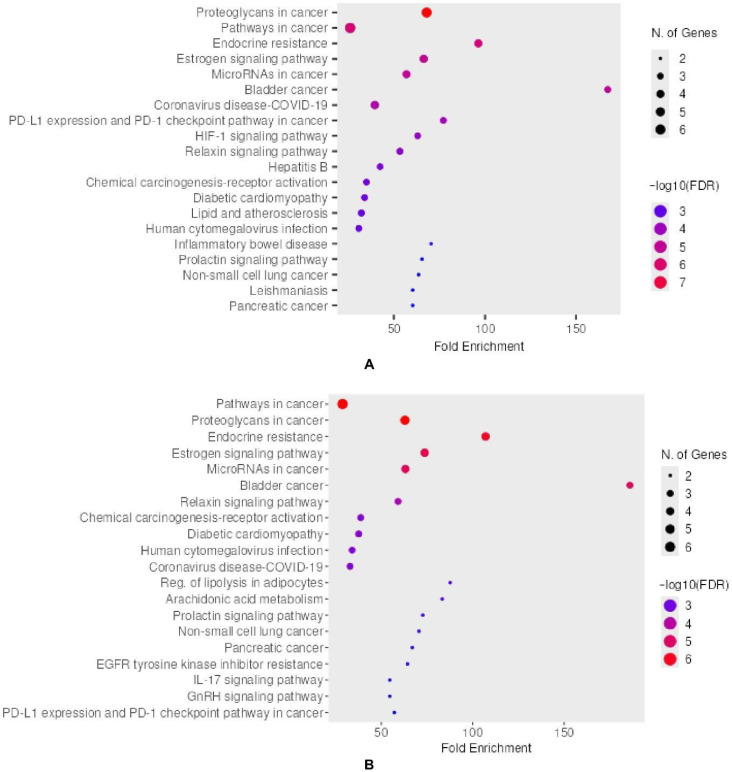
KEGG pathway enrichment analysis of the top 10 hub genes identified from PPI networks. (**A**) Significantly enriched pathways associated with colorectal cancer hub genes. (**B**) Significantly enriched pathways associated with ovarian cancer hub genes.

In colorectal cancer (Figure 7A), several enriched pathways reflect immune modulation and tumor microenvironment interactions, such as PD-L1/PD-1 checkpoint signaling and HIF-1 signaling, indicating potential involvement in immune escape and hypoxia-driven progression [70]. Additional enrichment in pathways linked to viral infections (such as hepatitis B and COVID-19) may reflect inflammatory or immune-related influences contributing to tumorigenesis [71,72]. Metabolic and cardiovascular-related pathways, including lipid and atherosclerosis and diabetic cardiomyopathy, further suggest systemic alterations influencing colorectal cancer development [73,74,75,76]. Enrichment in inflammatory bowel disease pathways aligns with known clinical associations between chronic inflammation and colorectal tumor progression [77].

Ovarian cancer (Figure 7B) also exhibited strong enrichment in cancer-related and hormone signaling pathways, including those related to prolactin, GnRH, and estrogen signaling. These pathways are reportedly involved in ovarian cancer [78,79]. Specific enrichment in EGFR tyrosine kinase inhibitor resistance highlights possible mechanisms of therapeutic resistance [80]. The identification of additional pathways, such as IL-17 signaling and arachidonic acid metabolism, suggests a unique inflammatory and lipid regulatory component in ovarian tumor biology [81]. Regulation of lipolysis in adipocytes may also point to metabolic adaptations relevant to the tumor microenvironment in ovarian cancer, as previously reported [82].

**Figure 8:**
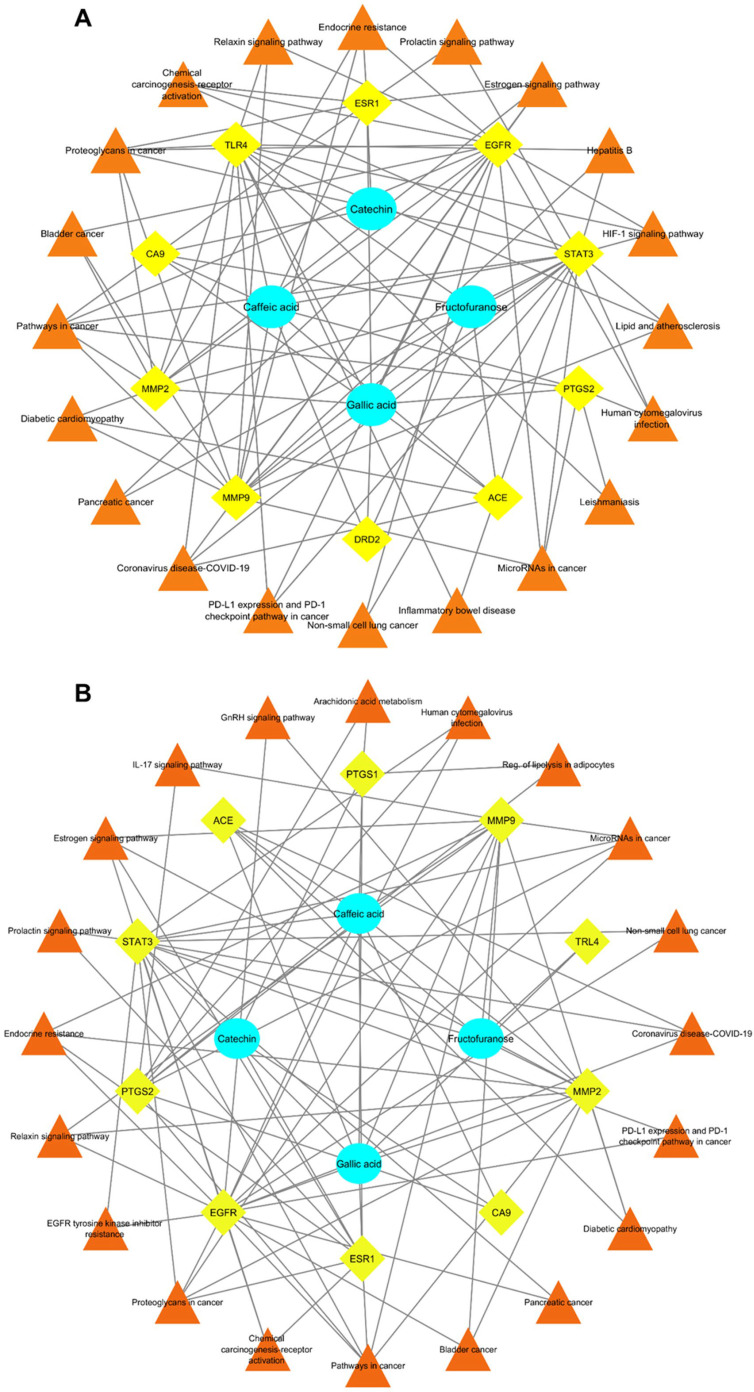
Compound–Target–Pathway interaction networks for the top hub genes involved in colorectal and ovarian cancer. (**A**) Network depicting the interactions among phytochemical compounds, shared hub gene targets, and enriched KEGG pathways in colorectal cancer. (**B**) Network representing compound–target–pathway relationships identified in ovarian cancer. Orange triangles indicate pathways, yellow diamonds represent gene targets, and blue circles correspond to phytochemical compounds.

To provide an integrative view of the molecular interactions involved in colorectal and ovarian cancers, compound–target–pathway networks were constructed (Figure 8). These networks illustrate the functional interplay between the selected bioactive compounds, the identified hub genes, and their enriched KEGG pathways. The visual layout emphasizes the centrality of key targets such as *EGFR*, *ESR1*, and *STAT3* (Figure 8). Figure 8 highlights the convergence of multiple compounds on shared oncogenic signaling cascades, particularly those involved in cancer proliferation, inflammation, and hormone regulation.

Importantly, crude plant extracts seldom act through a single bioactive constituent. Instead, multiple phytochemicals typically exert additive, antagonistic, or synergistic effects by modulating distinct yet interconnected signaling pathways. In the compound–target–pathway networks generated in this study, catechin, caffeic acid, and gallic acid converged on shared hub genes, such as *EGFR*, *ESR1*, and *STAT3*, while also influencing separate molecular pathways. This convergence supports the view that the biological activities of *R. ulmifolius* extract are likely the result of cooperative interactions among its constituents, consistent with the multi-compound synergy model widely recognized in phytomedicine [83].

#### 2.5.4. Molecular Docking of Core Targets and Compounds

**Figure 9:**
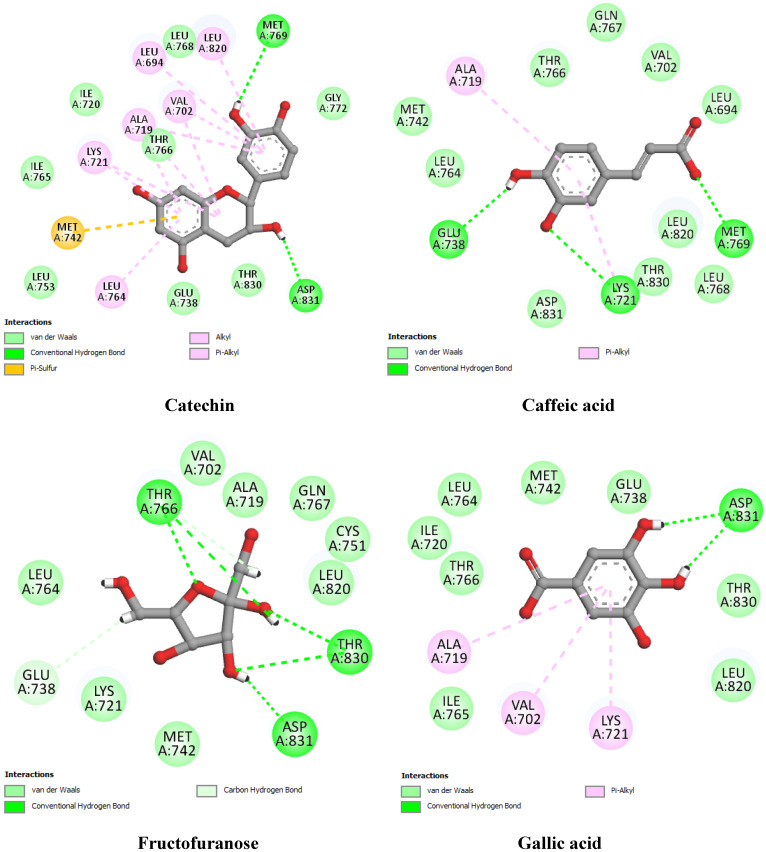
Two-dimensional interaction diagrams of docked compounds with EGFR (PDB ID: 1M17).

**Figure 10:**
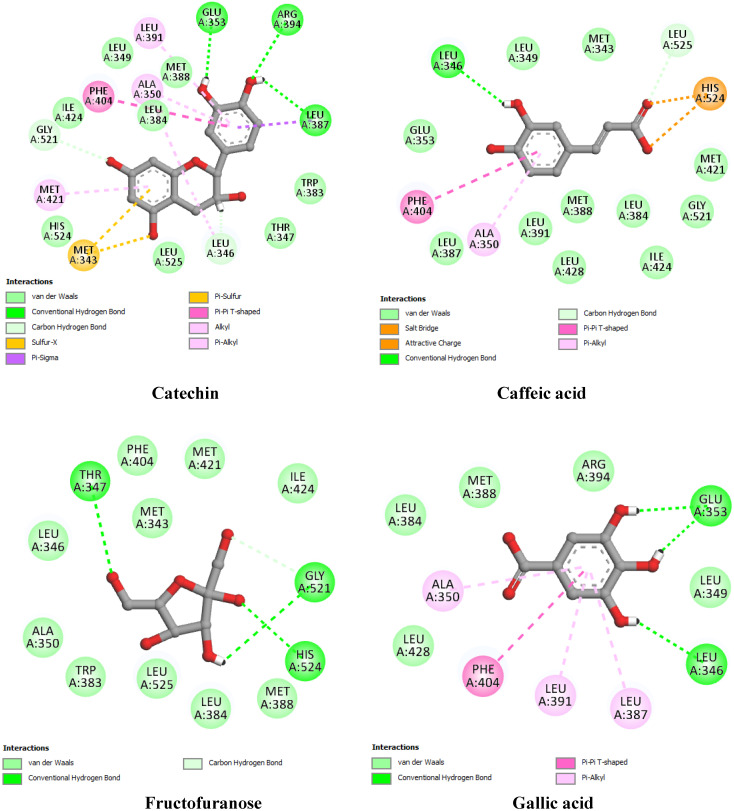
Two-dimensional interaction diagrams of docked compounds with Estrogen Receptor alpha (ESR1) (PDB ID: 1A52).

**Figure 11:**
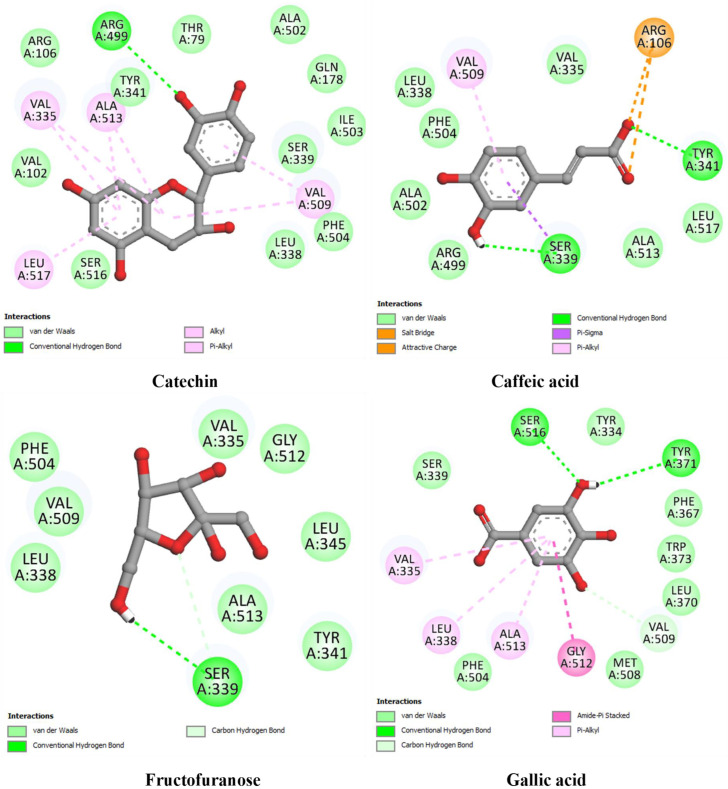
Two-dimensional interaction diagrams of docked compounds with Cyclooxygenase-2 (PTGS2) (PDB ID: 3LN1).

**Figure 12:**
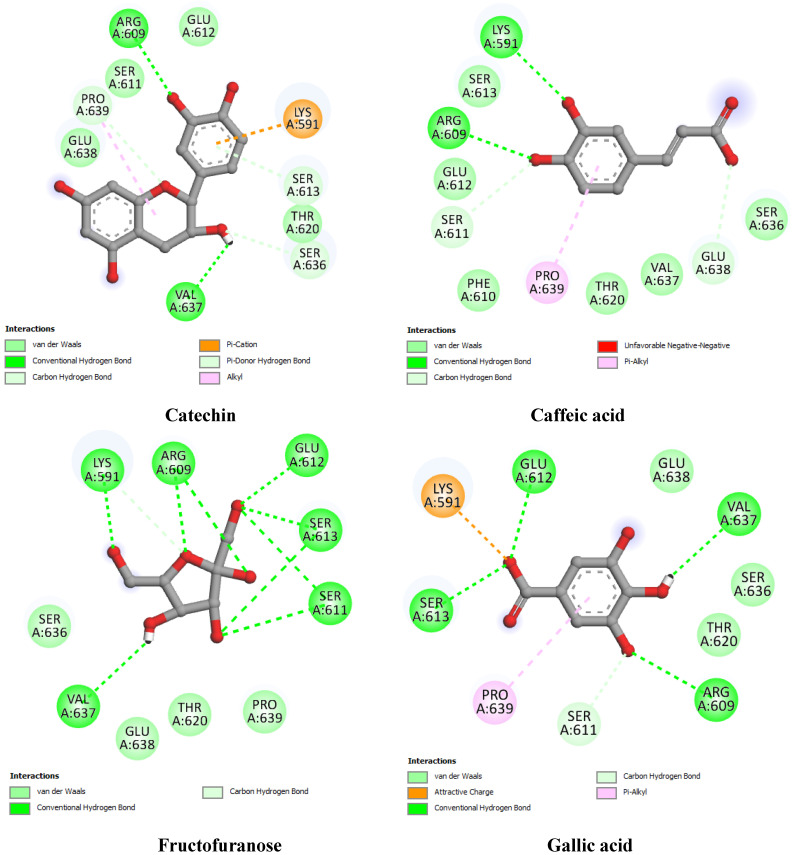
Two-dimensional interaction diagrams of docked compounds with Signal Transducer and Activator of Transcription 3 (STAT3) (PDB ID: 6NUQ).

To provide preliminary insights into the molecular mechanisms underlying the interaction between bioactive compounds and hub genes, we conducted a molecular docking analysis using EGFR, ESR1, PTGS2, and STAT3. These genes were selected based on their high degree values in the protein–protein interaction (PPI) network analysis, suggesting their central role in the biological processes associated with the disease. The binding affinity, hydrogen bonding interactions, and hydrophobic contacts were examined for each compound–target pair, with co-crystallized ligands or known inhibitors used as docking references (Table 5; Figure 9, Figure 10, Figure 11 and Figure 12).

For EGFR (PDB ID: 1M17) (Figure 9), the co-crystallized ligand 4-anilinoquinazoline showed a binding affinity of −8.01 kcal/mol and formed multiple hydrogen bonds with Met769, Gln767, and Thr830. Among the docked compounds, catechin exhibited the highest affinity (−7.56 kcal/mol), forming two hydrogen bonds with Met769 and Asp831, and engaging in stable hydrophobic contacts with key residues such as Ala719, Lys721, and Leu820. Caffeic acid and fructofuranose followed with binding energies of −6.85 and −6.12 kcal/mol, respectively. Gallic acid displayed the weakest affinity (−5.17 kcal/mol) among all EGFR–compound complexes.

Regarding ESR1 (PDB ID: 1A52) (Figure 10), the co-crystallized ligand estradiol demonstrated a binding energy of −7.80 kcal/mol and interacted via hydrogen bonds with His524, Arg394, and Glu353. Again, catechin had the highest docking score among the compounds (−7.32 kcal/mol), forming five hydrogen bonds and engaging hydrophobic contacts similar to the native ligand. Gallic acid (−6.04 kcal/mol) and caffeic acid (−5.91 kcal/mol) formed fewer interactions, while fructofuranose showed the lowest binding energy (−5.18 kcal/mol) for ESR1, consistent with its minimal hydrophobic interactions.

For PTGS2 (PDB ID: 3LN1) (Figure 11), the co-crystallized ligand celecoxib exhibited a strong binding affinity of −9.31 kcal/mol, stabilizing through several hydrogen bonds, including Arg499 and Ser339. Catechin emerged again as the top-performing compound (−7.89 kcal/mol), with hydrogen bonding to Arg499 and several hydrophobic contacts. Caffeic acid (−6.92 kcal/mol) and gallic acid (−6.33 kcal/mol) also interacted with catalytically relevant residues such as Tyr341 and Ser516. Fructofuranose exhibited the weakest binding affinity (−5.89 kcal/mol), lacking hydrophobic stabilization.

For STAT3 (PDB ID: 6NUQ) (Figure 12), the reference inhibitor Stattic was used for comparative docking, yielding a binding score of −7.41 kcal/mol. Interestingly, catechin surpassed this reference with a docking score of −8.12 kcal/mol, forming a rich network of hydrogen bonds, including Arg609, Ser613, and Lys591, along with a hydrophobic contact at Pro639. Caffeic acid and gallic acid also demonstrated moderate affinities (−7.08 and −6.75 kcal/mol, respectively), while fructofuranose presented the weakest interaction (−6.11 kcal/mol).

These results highlight catechin as the most promising compound, consistently demonstrating strong binding affinities across all core targets, often exceeding those of the reference ligands. Notably, its ability to form multiple hydrogen bonds and hydrophobic contacts appears critical for its enhanced interaction profile. Caffeic acid and gallic acid also displayed favorable interactions, albeit to a lesser extent. Fructofuranose, despite some polar interactions, generally exhibited lower binding energies and minimal hydrophobic engagement, indicating a weaker binding potential. Although catechin exhibited the most favorable docking scores, computational predictions alone cannot establish biological activity. Experimental validation, such as kinase inhibition or STAT3 transcriptional activity assays, as well as in vitro and in vivo experiments, are required to confirm these interactions.

In addition to their individual potential, *R. ulmifolius* phytochemicals (catechin, caffeic acid, and gallic acid) could be applied in combination strategies with existing chemotherapeutic agents such as difluoromethylornithine (DFMO), an irreversible inhibitor of ornithine decarboxylase [84], a key enzyme in polyamine biosynthesis that is frequently upregulated in colorectal and ovarian cancers [85,86], has shown clinical efficacy in colorectal cancer combination therapy with sulindac [87]. Combination therapy involving DFMO and natural compounds that target complementary pathways (e.g., STAT3, EGFR, or estrogen signaling) may enhance therapeutic efficacy through synergistic mechanisms by reducing drug resistance and enabling lower dosing of conventional agents, thereby minimizing toxicity [83,87]. Future studies should explore these interactions through co-treatment assays and mechanistic evaluations in preclinical models.

The study has several limitations that should be acknowledged. While informative, GC–MS profiling may underrepresent highly polar compounds such as polyphenols and flavonoids. Therefore, comprehensive profiling of the extract using complementary techniques such as HPLC or LC–MS/MS will be essential in future studies to confirm and extend these findings. In addition, the antifungal activity was assessed using the agar diffusion method, which provides a preliminary indication of growth inhibition but does not yield quantitative potency values. In future work, minimum inhibitory concentration (MIC) assays should be included to more precisely define antifungal efficacy. Furthermore, in this study, the focus was on evaluating the anticancer efficacy of the extract rather than its cytotoxicity profile. A key limitation, however, is that cytotoxicity was assessed exclusively in cancer cell lines without parallel testing in normal human cells, which limited conclusions about safety and selectivity. Although previous studies on *R. ulmifolius* extracts suggest low toxicity in non-tumorigenic cell lines [20,57,58], experimental confirmation is still required. Future work should therefore include cytotoxicity testing in normal human cells to better define the therapeutic concentration window and guide preclinical safety assessment. Finally, the proposed anticancer mechanisms of *R. ulmifolius* phytochemicals should be validated through in vitro studies and preclinical animal models using purified compounds, both individually and in combination therapies.

## 3. Materials and Methods

### 3.1. Chemical Composition of R. ulmifolius EtOAc Extract

The chemical composition of the EtOAc extract from *R. ulmifolius* leaves was characterized in our earlier studies [23]. The analysis revealed the presence of several bioactive secondary metabolites (Table 6), which are known for their diverse pharmacological properties, particularly antioxidant and antimicrobial activities.

### 3.2. Antifungal Activity

The fungal strains used in this study were *Fusarium oxysporum*, *Aspergillus niger* (GenBank Accession No. MH109542), *Aspergillus fumigatus* (MH109539), *Penicillium* sp., and *Candida albicans*. Filamentous fungi were cultured on Potato Dextrose Agar (PDA) medium and incubated at 28 °C for 14 days. Spore suspensions were prepared by scraping the culture surface with sterile physiological water. The resulting mixture was adjusted to an optical density of 0.15–0.2 at 650 nm, then diluted 1:10 with physiological water. In parallel, *C. albicans* was cultured on Sabouraud agar and incubated at 37 °C for 24–48 h. The yeast suspension was prepared in sterile physiological water and adjusted to 0.5 McFarland standard [88]. All fungal suspensions were inoculated on Sabouraud agar plates using sterile swabs. To create test zones, wells of 6 mm in diameter were punched into the agar. Each well was then filled with 40 μL of the test extracts prepared in DMSO at a concentration of 50 mg/mL. After allowing diffusion at 4 °C for 30 min, the plates were incubated at 28 °C for 48–72 h for molds and 24–48 h for *C. albicans*. Nystatin was used as the positive control [89]. Fungal strains were obtained from the Laboratory of BioEngineering, Higher National School of Biotechnology, Constantine, Algeria.

### 3.3. AChE and BChE Inhibition Assay

The cholinesterase inhibitory activity was assessed for both AChE and BChE enzymes. The enzymatic reaction was monitored using DTNB (5,5′-dithiobis-(2-nitrobenzoic acid) “as a chromogenic agent”). Each assay mixture consisted of 150 μL of sodium phosphate buffer (100 mM, pH 8.0), to which 10 μL of the extract, diluted in methanol at various concentrations, was added. Then, 20 μL of the enzyme solution, corresponding either to 5.32 × 10^−3^ U of AChE or 6.85 × 10^−3^ U of BChE, was introduced. The mixture was incubated at 25 °C for 15 min and then 10 μL of DTNB (0.5 mM) and 10 μL of the appropriate substrate (acetylthiocholine iodide at 0.71 mM for AChE, or butyrylthiocholine chloride at 0.2 mM for BChE) were added. Absorbance was measured at 412 nm using a microplate reader (Perkin Elmer, EnSpire, Waltham, MA, USA), immediately after substrate addition and again at 5, 10, and 15 min intervals to assess enzyme activity. Galantamine was employed as the standard reference inhibitor, and the inhibitory potency of the extracts was expressed in terms of IC_50_ values (μg/mL) [90].

### 3.4. Urease Inhibition Assay

The urease inhibitory activity was evaluated using the conventional indophenol-based colorimetric assay [91]. In a 96-well microplate, 10 μL of the extract prepared in methanol at varying concentrations were added to 25 μL of jack bean urease (5 U/mL). Subsequently, 50 μL of a 100 mM urea solution, 45 μL of a phenol reagent (8% phenol and 0.1% (*w*/*v*) sodium nitroprusside), and 70 μL of an alkaline reagent (containing 2.85% NaOH and 4.7% of chlorine available from NaOCl) were added in sequence. The plate was then incubated at 30 °C for 50 min, after which absorbance was recorded at 630 nm using a microplate reader (Perkin Elmer, EnSpire). Thiourea was used as the reference inhibitor compound, and the inhibitory effect was expressed as the IC_50_ value.

### 3.5. Cell Culture and Cytotoxic Assay

Human cancer cell lines A549 (lung adenocarcinoma), HT-29 (colorectal adenocarcinoma), and SK-OV-3 (ovarian cancer) were obtained from the American Type Culture Collection (ATCC, Manassas, VA, USA). A549 cells were maintained in RPMI medium (Gibco, Thermo Fisher Scientific, Waltham, MA, USA), while HT-29 and SK-OV-3 cells were cultured in DMEM (Gibco, Thermo Fisher Scientific). All media were enriched with 10% fetal bovine serum (FBS, Gibco), 2 mM L-glutamine, and antibiotics (100 U/mL penicillin and 0.1 mg/mL streptomycin). Cells were incubated at 37 °C in a humidified atmosphere containing 5% CO_2_ and subcultured when they became 80–90% confluent using trypsin–EDTA [54]. The antiproliferative effects of the EtOAc extract was assessed using the MTT assay. Briefly, 7.0 × 10^3^ cells were seeded in 96-well cell culture plates and allowed to adhere for 24 h. Thereafter, cells were exposed to increasing concentrations of the extract (0, 1, 5, 10, and 20 µg/mL) for 48 h. Wells treated with the vehicle alone were used as a negative control. After treatment, cells were rinsed with PBS and incubated for 3 h at 37 °C in the dark with the MTT reagent in the cell culture incubator. The formed formazan crystals were then solubilized using DMSO, and the absorbance was recorded at 540 nm [54,92]. Cell viability was determined using the following formula:

$$\%\;Cell\;viability=\frac{Absorbance\;of\;treated\;cells}{Absorbance\;of\;control\;cells}\times 100.$$

The IC_50_ values (concentration causing 50% inhibition of cell viability) were obtained from non-linear regression of dose–response curves. Cytotoxicity was categorized as: High (IC_50_ < 20 µg/mL), moderate (IC_50_ = 21–200 µg/mL), weak (IC_50_ = 201–500 µg/mL), and inactive (IC_50_ > 500 µg/mL), according to the National Cancer Institute (NCI) guidelines [54].

### 3.6. Statistical Analysis

All experiments were conducted in triplicate for each treatment (n = 3). Statistical analysis was performed using SPSS software (version 25.0) and GraphPad Prism 9. One-way ANOVA was applied to determine significant differences among groups, followed by Tukey’s HSD post hoc test for pairwise comparisons. A significance level of *p* < 0.05 was considered statistically significant. Results are presented as the mean ± standard error of the mean (SEM). The half-maximal inhibitory concentration (IC_50_) for cell growth was determined by fitting MTT assay data to dose–response curves, plotting percent growth inhibition against the log-transformed extract concentration in GraphPad Prism 9.

### 3.7. In Silico Study

#### 3.7.1. Prediction of *R. ulmifolius* Bioactive Compounds Gene Targets

The 2D structures of *R. ulmifolius* bioactive compounds identified by GC-MS (Table 6), caffeic acid, catechin, fructofuranose, and gallic acid, were retrieved in SMILES format from the PubChem database. Molecular targets for each compound were predicted using two complementary online tools: SwissTargetPrediction (http://www.swisstargetprediction.ch/, accessed on 20 August 2025) [93] and Similarity Ensemble Approach (SEA) (https://sea.bkslab.org/, accessed on 20 August 2025) [94]. Only human proteins were considered, and duplicate targets between the two databases were overlapped to produce a refined target list for each compound.

#### 3.7.2. Retrieval of Disease-Associated Genes

Colorectal and ovarian cancers were selected as the target diseases based on the in vitro cytotoxicity results, which demonstrated significant antiproliferative activity of EtOAc extract against HT-29 and SK-OV-3 cell lines. Disease-related target genes for colorectal and ovarian cancer were retrieved from the GeneCards (https://www.genecards.org/, accessed on 20 August 2025) [95] and OMIM (https://www.omim.org/, accessed on 20 August 2025) databases [96] using the keywords “colorectal cancer” and “ovarian cancer”. After merging and removing duplicates, the final disease-associated gene sets were established.

#### 3.7.3. Identification of Common Targets

Venn diagrams were constructed using Venny 2.1 (https://bioinfogp.cnb.csic.es/tools/venny/, accessed on 22 August 2025) to identify overlapping targets between each compound and the disease targets. In addition, gene targets common to at least two compounds were extracted for further analysis by overlapping the compound targets using Venny 2.1.

#### 3.7.4. Protein–Protein Interaction (PPI) Network Construction

The common targets for each cancer type were uploaded into the STRING database (https://string-db.org/, accessed on 22 August 2025) [97] to retrieve protein–protein interaction data with a minimum confidence score of 0.4 (medium confidence). The resulting PPI networks were exported and visualized using Cytoscape v3.10.2 [98]. Network parameters (e.g., number of nodes, edges, degree, clustering coefficient) were computed using the NetworkAnalyzer plugin of Cytoscape v3.10.2. Hub genes were identified based on node degree values.

#### 3.7.5. KEGG Pathway Enrichment Analysis

The top 10 hub genes identified for each cancer type were subjected to KEGG pathway enrichment analysis [69] using the ShinyGO v0.76.3 online platform (http://bioinformatics.sdstate.edu/go/, accessed on 22 August 2025) [99]. Significantly enriched pathways were identified using a threshold of *p* < 0.05. The results were visualized as dot plots and summarized into gene–pathway association tables for downstream network interpretation.

#### 3.7.6. Compound–Target–Pathway Network Construction

Based on the identified hub genes and enriched pathways, two integrated compound–target–pathway networks were built using Cytoscape, where nodes represented compounds, hub genes, or pathways, and edges denoted functional or molecular interactions.

#### 3.7.7. Molecular Docking Studies

Molecular docking was performed using AutoDock Vina software (version 1.1.2) [100]. Crystal structures of EGFR (PDB ID: 1M17), ESR1 (PDB ID: 1A52), PTGS2 (PDB ID: 3LN1), and STAT3 (PDB ID: 6NUQ) were downloaded from the protein data bank (PDB; https://www.rcsb.org/, accessed on 22 August 2025). The proteins were prepared by removing water molecules, adding polar hydrogen atoms, and assigning Kollman charges using AutoDock Tools (version 1.5.7). Energy minimization of the protein structures was performed using default parameters. All bioactive compounds were geometry-optimized and energy-minimized using MMFF94 force field in Avogadro software (version 1.2.0) prior to docking. Docking was carried out in a grid box centered on the active site of each protein, and the binding affinities were expressed in kcal/mol. A docking grid box was defined based on the coordinates of the co-crystallized ligand retrieved from the Protein Data Bank (PDB). A re-docking procedure was carried out to validate the docking protocol by calculating the root mean square deviation (RMSD) between the original and re-docked pose of the co-crystallized ligand. The docking results were ranked according to binding affinity values (kcal/mol), and the ligand–protein interactions were subsequently analyzed in both 2D and 3D representations.

## 4. Conclusions

The present study offers a comprehensive evaluation of the pharmacological potential of the EtOAc extract of leaves of *R. ulmifolius* Schott through a multidisciplinary approach combining in vitro bioassays and in silico analyses. The extract demonstrated marked antifungal activity against multiple pathogenic strains, along with moderate inhibitory effects on AChE, weaker inhibition of BChE, and a limited effect on urease. Furthermore, the extract exhibited potent cytotoxicity against HT-29 and SK-OV-3 cancer cell lines, suggesting its potential as a source of anticancer agents, especially colorectal and ovarian cancers. These biological effects could be attributed to the presence of key phytochemicals such as gallic acid, caffeic acid, and catechin. Through an integrative network pharmacology approach, common molecular targets shared between the selected compounds and disease-related genes were identified. PPI analysis revealed key hub genes such as *EGFR*, *ESR1*, *PTGS2*, and *STAT3*. Functional enrichment analysis, including KEGG pathway mapping, highlighted several cancer-associated signaling pathways, notably those involved in hormone signaling, inflammatory responses, and cell proliferation. Molecular docking predicted favorable binding affinities with cancer hub genes, particularly for catechin, suggesting a possible multi-target mode of action.

Moreover, the predicted convergence of several phytochemicals on overlapping cancer-related pathways highlights the possibility of synergistic interactions, which may contribute to the overall pharmacological activity of the extract. This synergy may be enhanced by combining these compounds with chemotherapeutics targeting distinct cancer pathways. Such combination therapy could improve efficacy, overcome therapy resistance, and reduce toxicity.

Future studies should aim to validate the identified *R. ulmifolius* phytochemicals, such as catechin, caffeic acid, and gallic acid, to confirm their interactions with cancer-related targets through dedicated biochemical and cellular assays. Moreover, in vivo studies will be required to confirm their efficacy and safety both individually and in combination. Such investigations will help to translate these findings into preclinical evidence.

## Figures and Tables

**Table 1:** Results of antifungal activity of *R. ulmifolius* ethyl acetate leaves extract.

	Zone of Inhibition (mm)				
	*C. albicans*	*A. niger*	*A. fumigatus*	*Penicillium* sp.	*F. oxysporum*
EtOAc extract	22.5 ± 0.7 ^d^	18.0 ± 1.0 ^f^	24.0 ± 0.6 ^d^	26.8 ± 1.3 ^c^	18.0 ± 1.0 ^e^
Nystatin	31.5 ± 0.8 ^b^	35.0 ± 0.9 ^a^	36.8 ± 1.2 ^a^	32.5 ± 0.6 ^b^	30.5 ± 0.3 ^b^

Values with different letters are significantly different at *p* < 0.05 according to Tukey’s HSD test.

**Table 2:** Inhibitory activity of EtOAc extract against AChE and BChE.

	IC_50_ (μg/mL)	
	BchE	AchE
EtOAc Extract	274.93 ± 2.32	92.94 ± 1.97
Galantamine	35.41 ± 0.52	7.14 ± 0.88

**Table 3:** Urease inhibitory activity of *R. ulmifolius* EtOAc extract.

	IC_50_ (µg/mL)
EtOAc Extract	262.60 ± 1.41
Thiourea	11.57 ± 0.68

**Table 4:** Cytotoxic effects of *R. ulmifolius* EtOAc extract against human cancer cell lines.

	HT-29	SK-OV-3	A549
**IC_50_ (µg/mL)**	2.41 ± 0.13	4.63 ± 0.26	>20

**Table 5:** Molecular docking results of four bioactive compounds (catechin, caffeic acid, fructofuranose, and gallic acid) against core cancer-related targets (EGFR, ESR1, PTGS2, and STAT3).

			Binding Energy (Kcal/mol)	Hydrogen Interactions (Distance Å)	Hydrophobic Interactions
**EGFR** **(1M17)**	Co-crystallized ligand	4-anilinoquinazoline	−8.01	Met769 (2.54), Gln767 (2.32), Thr830 (3.14)	Leu820(2), Ala719 (2), Leu764, Lys721 (2), Met769, Leu694
	Best docked compounds	Catechin	−7.56	Met769 (3.22), Asp831 (2.55)	Leu820, Leu694, Val702 (2), Ala719 (3), Lys721 (2), Leu764
		Caffeic acid	−6.85	Met769 (1.68), Lys721 (2.48), Glu738 (2.36)	Ala719, Lys721
		Fructofuranose	−6.12	Thr766 (2.55), Thr766 (2.82), Thr766 (2.96), Thr830 (1.98), Thr830 (2.87), Asp831 (2.15), Glu738 (2.69)	-
		Gallic acid	−5.17	Asp831 (2.44)	Ala719, Val702, Lys721
**ESR1** **(1A52)**	Co-crystallized ligand	Estradiol	−7.80	His524 (2.57), Arg394 (3.34), Glu353 (3.41)	Leu391, Phe404 (2), Ala350, Leu387, Let388, Leu384, Ile424, His524, Met421, Leu525, Leu346
	Best docked compounds	Catechin	−7.32	Arg394 (2.65), Glu353 (2.74), Gly512 (2.25), Leu346 (2.63), Leu387 (2.47)	Leu391, Ala350, Phe404, Met421, Leu356, Leu387
		Caffeic acid	−5.91	Leu346 (2.82), Leu525 (2.52)	Phe404, Ala350
		Fructofuranose	−5.18	Thr347 (3.16), Gly521 (2.50), Gly521 (2.82), His524 (2.36)	-
		Gallic acid	−6.04	Glu353 (2.51), Glu353 (2.58), Leu346 (3.36)	Ala350, Phe404, Leu391, Leu387
**PTGS2** **(3LN1)**	Co-crystallized ligand	Celecoxib	−9.31	Arg499 (2.30), Gln178 (2.48), Leu338 (2.51), Ser339 (2.71), Arg106 (3.42)	Ser339, Val509, Leu517, Ala513, Val335 (2), Gly512, Tyr371, Trp373, Leu370
	Best docked compounds of olive oil	Catechin	−7.89	Arg499 (2.68)	Val335 (2), Ala513 (2), Leu517, Val509 (2)
		Caffeic acid	−6.92	Ser339 (2.48), Tyr341(2.69)	Val509, Ser339
		Fructofuranose	−5.89	Ser339 (3.48), Ser339 (2.61)	-
		Gallic acid	−6.33	Ser516 (2.49), Tyr371 (2.62), Val509 (2.72)	Val335, Leu338, Ala513, Gly512
**STAT3** **(6NUQ)**	Reference inhibitor	Stattic	−7.41	Ser611 (1.84), Ser611 (2.50), Glu612 (2.45), Ser613 (2.04), Ser613 (2.46), Lys591 (3.08),	Pro639
	Best docked compounds	Catechin	−8.12	Arg609 (2.25), Arg609 (2.51), Pro639 (2.44), Val637 (2.49), Ser636 (2.53), Ser613 (2.83), Lys591(2.36)	Pro639
		Caffeic acid	−7.08	Ser611 (2.54), Arg609 (2.78), Lys591 (2.92), Glu638 (3.43)	Pro639
		Fructofuranose	−6.11	Ser611 (3.25), Ser611 (2.55), Ser613 (2.95), Ser613 (2.46), Glu612 (2.38), Arg609 (2.74), Arg609 (2.69), Lys591 (2.60), Lys591 (2.48), Val637 (2.65)	-
		Gallic acid	−6.75	Ser611 (2.44), Ser611 (3.12), Ser613 (2.74), Glu612 (2.52), Arg609 (3.45), Val637 (2.35)	Pro639

**Table 6:** List of compounds in the crude EtOAc extract identified by GC-MS analysis. RT = retention time; RI = Kovats retention index.

Compound	RT	RI
D-(-)-Fructofuranose	12.669	1831
Gallic acid	13.28	1974
Caffeic acid	13.916	2148
Catechin	18.28	2900

## Data Availability

The data are contained within the article. Any additional data can be requested from the corresponding authors.
